# Symptomatic Bochdalek Hernia in Pregnancy: A Rare Case Report

**DOI:** 10.1155/2017/2862149

**Published:** 2017-10-16

**Authors:** Selçuk Yetkinel, Çağhan Pekşen, Remzi Kızıltan

**Affiliations:** ^1^Department of Gynecology and Obstetrics, Agri State Hospital, 04000 Ağrı, Turkey; ^2^Department of Surgery, School of Medicine, Van Yüzüncü Yıl University, 65090 Van, Turkey

## Abstract

**Introduction:**

Symptomatic Bochdalek hernia in pregnancy is quite rare. To the best of our knowledge, there are a total of 44 cases reported in the literature between 1959 and 2016 (Hernández-Aragon et al., 2015; Koca et al., 2016). Difficulty and delay in diagnosis may lead to life-threatening complications.

**Case Report:**

We report a case of Bochdalek hernia during the 30 gestational weeks' pregnancy in whom pregnancy continued after surgical repair which resulted in term birth.

**Discussion:**

Bochdalek hernia is diagnosed with an incidence of 1 in 2200–12500 live births, while symptomatic diaphragm hernia is much less in adults. The actual incidence of diaphragmatic hernias during pregnancy is still unknown. Symptoms may include abdominal distension, recurrent abdominal pain, nausea, vomiting, inability to defecate, dyspnea, and chest pain. The patient with diaphragmatic hernia may be asymptomatic until the late weeks of gestation, as in our case, or herniation may occur during advanced gestational weeks with increased intraabdominal pressure.

**Conclusion:**

In conclusion, diagnosis of the diaphragm hernia during pregnancy is very rare. Diagnosis is rarer in symptomatic patients due to its rarity and the duration of diagnosis may, therefore, be delayed. Diaphragm hernia should be kept in mind in symptomatic patients due to its high maternal and fetal mortality rates.

## 1. Introduction

Symptomatic Bochdalek hernia in pregnancy is quite rare. To the best of our knowledge, there are a total of 44 cases reported in the literature between 1959 and 2016 [1, 2]. Difficulty and delay in diagnosis may lead to life-threatening complications. If strangulation develops, the mortality can be as high as 32% [3]. The diaphragmatic hernia seen in adults usually results in an anatomic disorder already present in the diaphragm, a condition which ruptures due to conditions that increase intraabdominal pressure such as trauma, pregnancy, or birth. Therefore, women with diaphragmatic hernia may not be diagnosed until pregnancy [4].

Herein, we report a case of a woman with Bochdalek hernia during the 30 gestational weeks' pregnancy in whom pregnancy continued after surgical repair which resulted in term birth.

## 2. Case Report

**Figure 1:**
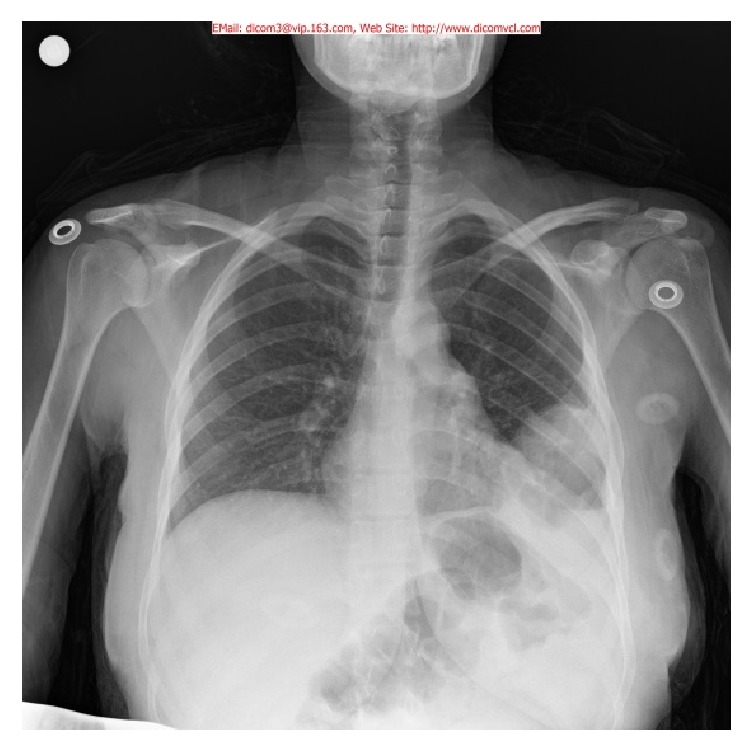
A herniated bowel loop in posteroanterior chest X-ray.

A 23-year-old, 29/6 gestational week according to the last menstrual period (G2/P1) pregnant woman was referred to Agri State Hospital Emergency Medicine Department with a complaint of abdominal pain radiating to the left side for one day. She previously underwent cesarean section due to breech presentation of her first baby three years ago. Vital signs were as follows: pulse rate, 110 beats/min, blood pressure, 110/60 mmHg, O_2_ saturation, 96%, and body temperature, 36.8°C. Physical examination revealed that abdomen was normal without any defense or rebound. No contraction was detected manually. The height of fundus was measured as 30 cm. Her lung sounds were also normal in the right hemithorax, whereas the sounds decreased in the left hemithorax bases. Laboratory results were as follows: white blood count of 13*∗*10^9^/L and hemoglobin of 11.2 g/dL. There was no abnormality in the other laboratory tests and complete urinalysis. Her medical history revealed no gas or stool discharge since the previous day. The fetus was positioned at the vertex position on obstetric ultrasonography, and the measurements were consistent with the gestational week. Amniotic fluid index was normal and cervical length was measured as 36 mm. No cervical opening and bleeding were observed in the pelvic examination. Nonstress test (NST) result was reactive. The patient was hospitalized for close monitoring. Repeated physical examination showed defense and rebound in the upper quadrant of the abdomen. Therefore, abdominal ultrasonography was planned. However, the abdomen was unable to be evaluated clearly due to intense gas. Respiratory sounds were slightly heard according to ultrasonography results of the left hemithorax. The posteroanterior chest X-ray with an abdominal shield was performed. On chest X-ray, diaphragm herniation in left hemithorax and herniated bowel loop were detected (Figure 1).

**Figure 2:**
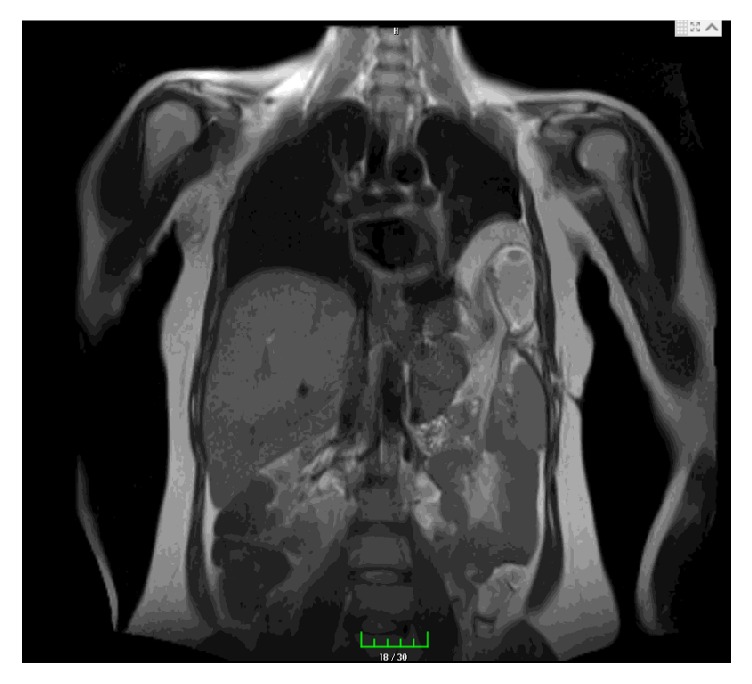
Thoracoabdominal magnetic resonance imaging showing irreducible Bochdalek hernia.

Based on her clinical and imaging findings, she was diagnosed with diaphragm hernia and was scheduled for surgery. The initial dose of betamethasone was administered to the patient in the 30th gestational week of pregnancy to provide pulmonary maturation of the infant with the possibility of preterm delivery. The patient was then transferred to a tertiary hospital, Van Yüzüncü Yıl University, Department of General Surgery, as she might be required to undergo the operation and there might be a necessity for neonatal intensive care unit. According to the thoracoabdominal magnetic resonance imaging result in the general surgery clinic, the patient underwent operation with the diagnosis of irreducible Bochdalek hernia (Figure 2).

**Figure 3:**
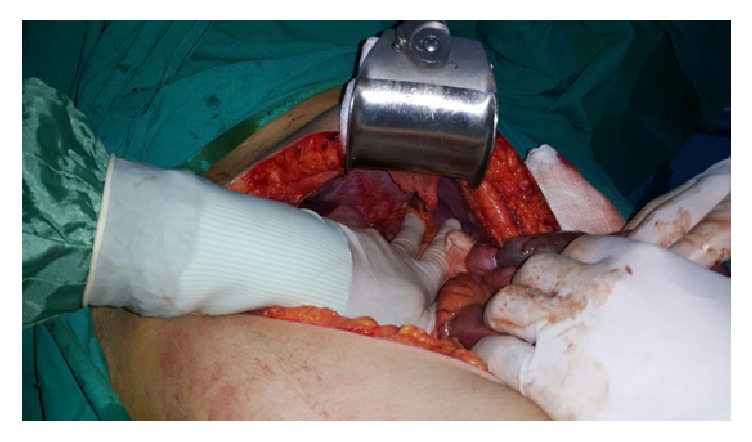
An intraoperative image of diaphragmatic defect.

Once entered into the abdomen with a median incision above the umbilicus, a 4 × 4 cm defect in the diaphragm was detected from which the transverse colon herniated into the thorax. The reduction of the transverse colon and the primer repair of diaphragm were then performed (Figure 3). Circulatory defect was not detected in the transverse colon segment. A resection of the bowel segment was not performed.

The patient was followed up in the general surgery ward for three days and had gas and stool discharge on the first postoperative day. Oral intake tolerance was good. At 31/3 gestational week, she had no additional complaints and was discharged on the fourth postoperative day. However, during routine follow-up, she was rehospitalized due to contractions at 37/5 gestational week. Based on her medical history of cesarean section previously, she gave a live term birth with a cesarean section. The infant had an Apgar score of 3 and 10 with a birth weight of 3,650 g. Both the patient and the infant were discharged on the second postoperative day uneventfully.

## 3. Discussion

Bochdalek hernia, the most common congenital diaphragm hernia in 95% of cases, is followed by Morgagni hernia and esophageal hernia [5]. Bochdalek hernia is diagnosed with an incidence of 1 in 2200–12500 live births, while symptomatic diaphragm hernia is much less in adults [6]. The actual incidence of diaphragmatic hernias with symptoms diagnosed during pregnancy is still unknown. Crump described the first Bochdalek hernia in pregnancy in the literature in 1911 [7]. To the best of our knowledge, there have been a total of 44 cases in pregnancy reported in the literature since 1959 [1, 2]. The symptoms may vary according to the organs herniated and the location covered in the hemithorax. Gastrointestinal symptoms may include abdominal distension, recurrent abdominal pain, nausea, vomiting, and inability of gas and stool discharge. In addition, respiratory symptoms may include dyspnea and chest pain. As in our case, patients with diaphragmatic hernia may be asymptomatic until advanced gestational weeks due to increased intraabdominal pressure or herniation may develop during advanced gestational weeks due to increased intraabdominal pressure. Due to pregnancy, the diagnosis may be difficult and fatal maternal and fetal complications may develop due to strangulation [8].

For diagnosis of Bochdalek hernias, chest radiographs, ultrasound, computed tomography (CT), and magnetic resonance imaging (MRI) can be used. Chest X-ray examination can be used safely even during pregnancy with a sensitivity of 70%. Therefore, normal view of chest X-ray cannot exclude herniation. CT is gold-standard imaging in elective and emergency cases and is useful to show defects in the diaphragm and thickening of the diaphragm and the herniated organs. MRI may be applicable in patients who are hemodynamically stable and cannot undergo computed tomography (e.g., pregnant or allergic to IV contrast) [9].

During pregnancy, the management of Bochdalek hernia can be difficult. Follow-up and treatment at a tertiary healthcare center would be more suitable. If the pregnant woman is in between 24–34 gestational weeks, it is appropriate to initiate steroid treatment for lung maturation and perform nasogastric decompression to save time until transfer of the patient and surgery are planned.

Treatment is planned based on the symptoms of the patient and gestational week. In the asymptomatic patients, elective surgery should be performed in the first or second trimester. In the third trimester, elective surgery also should be performed after fetal lung maturity is documented, with simultaneous cesarean section. Surgical treatment should be planned in emergency cases without considering the gestational week, as in our case in whom symptoms of obstruction developed [10].

Transabdominal and transthoracic approaches are also available in repair of diaphragmatic hernia. However, transabdominal approach is at the forefront plan for its ease of surgical technique [11]. Laparoscopic method is at the forefront of elective diaphragmatic hernia repair, whereas its use for emergency cases is uncommon, although it may be performed [12]. Primary repair of the diaphragm opening is the essential surgical method and the risk of its recurrence ranges from 10 to 40%. Although the use of synthetic or biological patches has become widespread in the current surgical approach, the superiority of primary repair has not been clarified yet [11].

Review of the literature reveals that the complication rates are more frequent in the third trimester of pregnancy: preterm labor was observed in 24% of these cases, while fetal death was observed in 13% [10]. In our case, the patient underwent surgery before preterm delivery, and follow-up was performed after surgery. The patient gave a live term birth. It should be kept in mind that the rate of mortality increases when the obstruction symptoms begin, possibly due to maternal respiratory distress, perforated gastrointestinal system, cardiogenic shock, and fetal hypoxia and acidosis [10].

## 4. Conclusion

In conclusion, diagnosis of the diaphragm hernia during pregnancy is very rare. Diagnosis is rarer in symptomatic patients due to its rarity and the duration of diagnosis may, therefore, be delayed. Diaphragm hernia should be kept in mind in symptomatic patients due to its high maternal and fetal mortality rates.
